# Myxopyronin B inhibits growth of a Fidaxomicin-resistant *Clostridioides difficile* isolate and interferes with toxin synthesis

**DOI:** 10.1186/s13099-021-00475-9

**Published:** 2022-01-06

**Authors:** Madita Brauer, Jennifer Herrmann, Daniela Zühlke, Rolf Müller, Katharina Riedel, Susanne Sievers

**Affiliations:** 1 Institute of Microbiology, University of Greifswald, Greifswald, Germany; 2 Helmholtz Institute for Pharmaceutical Research Saarland (HIPS)—Helmholtz Centre for Infection Research (HZI) and Department of Pharmacy, Saarland University, Saarbrücken, Germany; 3 German Center for Infection Research (DZIF), Braunschweig, Germany

**Keywords:** RNA polymerase inhibitors, Rifaximin, Fidaxomicin, Myxopyronin B, *Clostridioides difficile*, Antibiotic therapy, Antibiotic resistance, Mass spectrometry, Toxins

## Abstract

The anaerobic, gastrointestinal pathogen *Clostridioides difficile* can cause severe forms of enterocolitis which is mainly mediated by the toxins it produces. The RNA polymerase inhibitor Fidaxomicin is the current gold standard for the therapy of *C. difficile* infections due to several beneficial features including its ability to suppress toxin synthesis in *C. difficile*. In contrast to the Rifamycins, Fidaxomicin binds to the RNA polymerase switch region, which is also the binding site for Myxopyronin B. Here, serial broth dilution assays were performed to test the susceptibility of *C. difficile* and other anaerobes to Myxopyronin B, proving that the natural product is considerably active against *C. difficile* and that there is no cross-resistance between Fidaxomicin and Myxopyronin B in a Fidaxomicin-resistant *C. difficile* strain. Moreover, mass spectrometry analysis indicated that Myxopyronin B is able to suppress early phase toxin synthesis in *C. difficile* to the same degree as Fidaxomicin. Conclusively, Myxopyronin B is proposed as a new lead structure for the design of novel antibiotics for the therapy of *C. difficile* infections.

**Supplementary Information:**

The online version contains supplementary material available at 10.1186/s13099-021-00475-9.

## Introduction

The anaerobic, spore-forming pathogen *Clostridioides* *difficile* infects the intestine of higher mammals, especially of humans and pigs, after colonization resistance that is generally provided by the microbiota is disrupted, e.g. after antibiotic therapy [1–4]. *C.* *difficile* infections (CDI) are routinely treated with antibiotics, such as Fidaxomicin and Vancomycin, which usually stop acute infections [3]. In severe cases, fecal microbiota transplantation is a possible alternative although still critically discussed [5]. However, recurrence rates due to remaining spores and biofilm-associated cells are substantially high and a major issue in the context of CDI [6, 7]. RNA polymerase inhibitors have been among the first antibiotics approved for clinical therapy [8], and they are still in use for the treatment of severe bacterial infections such as tuberculosis, peptic ulcer disease, and traveler’s diarrhea [9]. Also in CDI, RNA polymerase inhibitors are frequently used. Rifaximin, a derivative of Rifamycin, is occasionally used as chaser post-vancomycin treatment for severe and recurrent forms of CDI [10]. Moreover, the macrocyclic antibiotic Fidaxomicin is considered as the current gold standard in CDI therapy [3, 11]. Fidaxomicin’s superiority in CDI therapy over other antibiotics is mostly ascribed to its relative selectivity for *C. difficile,* providing the microbiota a greater chance to recover and re-establish colonization resistance, and thus lowering rates of reinfection and recurrence [6, 7]. Furthermore, Fidaxomicin proved to have a negative impact on *C. difficile’s* toxin production, sporulation and spore germination [12–15]. In contrast to the Rifamycins, Fidaxomicin and other RNA polymerase inhibitors, such as the myxobacterial natural products Myxopyronins, Corallopyronin and Ripostatin, target the RNA polymerase switch region, which is required for the opening of the RNA:DNA clamp [16, 17]. Thereby, they interfere with the transcriptional process at an earlier stage than Rifamycins and do not show cross-resistance with Rifamycin and its derivatives [16–19]. Moreover, biotechnological production of Myxopyronin has become feasible due to a heterologous expression system enabling stable and high-yield fermentation of this compound [20]. Recently, the isolation of a *C. difficile* strain with drastically reduced Fidaxomicin susceptibility due to a mutation in the RNA polymerase switch region was reported, which is of high concern [21]. In view of this, the alpha-pyrone antibiotic Myxopyronin B [22], which was previously shown to be active against *C. difficile* [17], might be a potential lead structure for the design of alternative CDI antibiotics. Based on structural analyses of the antibiotics’ binding site [19, 23, 24], cross-resistance with Fidaxomicin is not expected, but still needs to be experimentally proven. This study confirmed the antimicrobial activity of Myxopyronin B against a Fidaxomicin-resistant *C. difficile* strain, mapped the proteome stress signature that is caused by Myxopyronin B in *C. difficile* compared to other RNA polymerase inhibitors, and investigated the effect of Myxopyronin B on toxin synthesis.

## Materials and methods

### Antibiotics and strains

*C. difficile* strains of human origin, namely 630 (DSM 27543, ribotype (RT) 012), 1780 (DSM 1296, RT001), R20291 (DSM 27147, RT027) and Goe-91 (DSM 105001, RT007/014/025) [21], and commensal bacteria *Clostridium scindens* VPI13733 (DSM 5676), *Lactobacillus casei* (DSM 20011), *Bifidobacterium longum* subsp. *infantis* (DSM20088), *Terrisporobacter* *sp. *CCk3R4-PYG-107 (DSM 29186), *Bacteroides fragilis* VPI 2553 (DSM 2151) and *Bacteroides thetaiotaomicron* WAL 2926 (DSM 2255) were obtained from the German Collection of Microorganisms and Cell Cultures (DSMZ; Braunschweig, Germany). Porcine *C. difficile* isolates 11S0047 (RT126) and 12S0133 (RT78) were obtained from the group of Christian Seyboldt (FLI, Jena, Germany) [25]. Myxopyronin B was isolated from *Myxococcus fulvus* Mxf50 [22] and it was provided by J. Herrmann and R. Müller (HZI-HIPS). Rifaximin and Fidaxomicin were obtained from Sigma Aldrich (St. Louis, Missouri, USA) and Selleckchem (Houston, Texas, USA). All antibiotics were dissolved in dimethyl sulfoxide (DMSO; Sigma Aldrich, St. Louis, Missouri, USA).

### Determination of minimal inhibitory concentrations

The minimal inhibitory concentrations of Rifaximin, Fidaxomicin and/or Myxopyronin B were determined in serial broth dilution assays in BHIS medium (Brain Heart Infusion broth, 5% yeast extract, 1% L-cysteine, 0.1% vitamin K, 0.5% hemin(chloride)) after 24 h of growth under anaerobic conditions (98% N_2_, 2% H_2_)*.* All minimal inhibitory concentrations assays were performed using at least three biological replicates per bacterial strain.

### Mass spectrometry (MS) analysis

*C. difficile* 630 was grown in CDMM medium [26] to mid-exponential phase and stressed with sublethal concentrations of Rifaximin (1.75 ng/ml), Fidaxomicin (6 ng/ml), and Myxopyronin B (500 ng/ml). Cells were grown in the presence of the antibiotics for further 90 min. Antibiotic-treated cells and cells grown with 0.06% (v/v) DMSO only were harvested for protein extraction. The cells were lyzed by bead beating in a FastPrep-25 homogenizer (MP Biomedicals, Santa Ana, California, USA; three cycles at 6.5 m/s à 30 s). Glass beads and cell debris were removed by two centrifugation steps at 15,000 rpm and 4 °C for 10 min and 20 min, respectively. Protein extracts were stored at − 70 °C. Protein concentrations were determined using Roti®-Nanoquant (Roth, Karlsruhe, Germany) according to the manufacturer’s instructions and 50 µg of each protein extract were reduced with 10 mM dithiothreitol (Sigma Aldrich, St. Louis, USA), alkylated with 20 mM iodoacetamide (Sigma Aldrich, St. Louis, USA) and acidified with phosphoric acid (Carl Roth®, Karlsruhe, Germany). Samples were loaded on S-traps (ProtiFi, Farmingdale, NY, USA) and proteins were digested with trypsin (Promega, Madison, USA) according to the manufacturer’s recommendations for 3 h. Trypsinized peptides were purified and fractionated by a high pH reversed-phase workflow on self-packed C_18_ columns as done previously [27]. MS samples were analyzed on a Q Exactive™ HF Hybrid Quadrupole-Orbitrap™ Mass Spectrometer coupled to an EASY nLC 1200 HPLC (Thermo Fisher Scientific, Waltham, Massachusetts, USA). Peptides were loaded onto an analytical column containing self-packed C_18_ reversed-phase material (3 µm, Dr. Maisch, Germany) with integrated emitter (100 µm × 20 cm). Peptides were eluted from the column using an 85 min gradient from 5 to 50% of acetonitrile, 0.1% acetic acid with a constant flow rate of 300 nL/min. Full survey scans were performed with a resolution of 60,000 in the range of 333 – 1650 m/z. Subsequently, MS/MS scans were performed for the fifteen most abundant precursor ions per scan cycle excluding unassigned charge states and singly charged ions. Dynamic exclusion was enabled for 30 s. Internal lock mass calibration was applied to a lock mass of *m/z* 445.12003.

### MS data analysis

LC–MS/MS data were searched against a strain specific protein database (3762 entries, obtained from Uniprot on March 15th, 2021 (UP000001978)) using the Andromeda based search engine MaxQuant ([28]; version 1.6.17.0). Common contaminants and reverse sequences were added by the MaxQuant software and the following parameters were set: Trypsin was chosen as digestion enzyme assuming a maximum of two missed cleavages. Oxidation of methionine was allowed as variable modification and carbamidomethylation of cysteine was selected as fixed modification. The false-discovery rate was set to 0.01. For protein identification default parameters were chosen. Label-free protein quantification was performed based on unique and razor peptides with a minimum ratio count of 2. Match between runs was enabled within each sample group. At least two unique peptides in at least two out of three biological replicates were required for *C. difficile* proteins to be identified and quantified. Log2 fold changes were calculated based on averaged LFQ intensities. For identification of significantly changed protein intensities the R package DEqMS [29] was used with an adjusted p value ≤ 0.05 considered as significant for proteins, which revealed a log2 fold change ≥ 1. Functional annotations of proteins were obtained and modified from the PathoSystems Resource Integration Center (PATRIC) on patricbrc.org [30]. Protein localizations were obtained from PSORTb [31]. Operon structures were obtained from Microbes online [32]. MS data were visualized using the R packages “ggvenn” [33] and “pheatmap” [34].

### Western blot analysis

50 µg of protein samples were separated by SDS PAGE on 8% SDS gels for 3 h at 80 V. Proteins were blotted on polyvinylidene fluoride membranes (Merck Millipore, Burlington, USA) for 1.5 h at 100 V. Blotted membranes were blocked in 5% skim milk in Tris-buffered saline (TBS; 6 g/l Tris, 9 g/l NaCl, pH 7.6) and incubated with primary antibodies against toxin A (1:5000, tgcBiomics, Bingen, Germany) or toxin B (1:5000, provided by Ralf Gerhardt, Hannover Medical School, Hannover, Germany) [35] at 4 °C overnight. Subsequently, membranes were washed three times in TBST (TBS, 0.1% Tween 20) and incubated with the appropriate secondary antibody (anti-mouse for toxin A, anti-rabbit for toxin B, Sigma Aldrich, St. Louis, USA) for 1 h at room temperature. After three washing steps with dH_2_O and 30 min of incubation in alkaline phosphatase buffer (AP buffer; 100 mM Tris, 100 mM NaCl, 5 mM MgCl_2_, pH 9.5), toxin signals were detected with 400 nM nitro blue tetrazolium chloride and 500 nM 5-bromo-4-chloro-3-indolyl phosphate (both solved in dimethylformamide) in AP buffer. Blots were scanned and signals were quantified using ImageJ [36].

### Statistical analysis

Statistical differences were determined by FDR-adjusted t-testing with the R package “RStatix” [37]. An adjusted p value of ≤ 0.05 was considered significant and marked with an asterisk.

## Results

### No cross-resistance with Myxopyronin B in a Fidaxomicin-resistant *C.* *difficile* isolate

As a starting point, the sensitivity of five *C. difficile* strains to the reference antibiotic Rifaximin and the natural product Myxopyronin B was determined in serial broth dilution assays. Strains of human or porcine origin, which belonged to five different ribotypes, were used to account for variation between different *C. difficile* strains. Minimal inhibitory concentrations of Myxopyronin B against *C. difficile* strains ranged from 0.125 to 8 µg/ml whereas minimal inhibitory concentrations of Rifaximin against *C. difficile* ranged from 0.002 to 0.004 µg/ml (Table 1). Subsequently, the minimal inhibitory concentrations of Rifaximin, Fidaxomicin and Myxopyronin B against the broadly used strain *C. difficile* 630 and the Fidaxomicin-resistant *C. difficile* strain Goe-91, which was recently isolated from a CDI patient [21], were evaluated. Both strains were tested sensitive to Rifaximin (Table 2). The Fidaxomicin-resistant strain Goe-91, as expected, demonstrated a high minimal inhibitory concentration value of 128 μg/ml towards Fidaxomicin, whereas strain 630 was susceptible to this antibiotic; however, an identical minimal inhibitory concentration value of 8 μg/ml for Myxopyronin B was seen in both strains, indicating that there is no cross-resistance between Fidaxomicin and Myxopyronin B. Additionally, the susceptibility testing of six anaerobic intestinal commensals to Myxopyronin B suggested that these bacteria are comparatively less sensitive than *C. difficile* apart from *Clostridium scindens* (Table 3).

**Table 1:** Minimal inhibitory concentrations Rifaximin and Myxopyronin B against *C. difficile*

	630	1780	R20291	RT126	RT78
Rif	0.002	0.004	0.004	0.004	0.004
MyxB	8	0.125	4	0.5	0.5

Minimal inhibitory concentrations of the reference antibiotic Rifaximin (Rif) and the natural product Myxopyronin B (MyxB) were determined against five different *C. difficile* strains in serial broth dilution assays after 24 h of growth in BHIS. Concentrations are given in µg/ml and are means of three biological replicates.

**Table 2:** Minimal inhibitory concentrations of Rifaximin, Fidaxomicin and Myxopyronin B for *C. difficile* strains 630 and Goe-91

	630	Goe-91
Rif	0.002	0.002
Fid	0.016	128
MyxB	8	8

Minimal inhibitory concentrations of the reference antibiotics Rifaximin (Rif) and Fidaxomicin (Fid) and the natural product Myxopyronin B (MyxB) were determined against *C. difficile* strains 630 and Goe-91 in serial broth dilution assays after 24 h of growth in BHIS. Concentrations are given in µg/ml and are means of three biological replicates.

**Table 3:** Minimal inhibitory concentrations of Myxopyronin B for six commensal intestinal anaerobes

	*Lactobacillus casei*	*Bifidobacterium longum*	*Clostridium scindens*	*Terrisporobacter sp.*	*Bacteroides fragilis*	*Bacteroides thetaiotaomicron*
MyxB	> 64	> 16	2	16	> 16	64

Minimal inhibitory concentrations of Myxopyronin B (MyxB) were determined against six commensal intestinal anaerobes in serial broth dilution assays after 24 h of growth in BHIS. Concentrations are given in µg/ml and are means of three biological replicates.

### Comparative stress response patterns of three RNA polymerase inhibitors in *C. difficile*

**Fig. 1:**
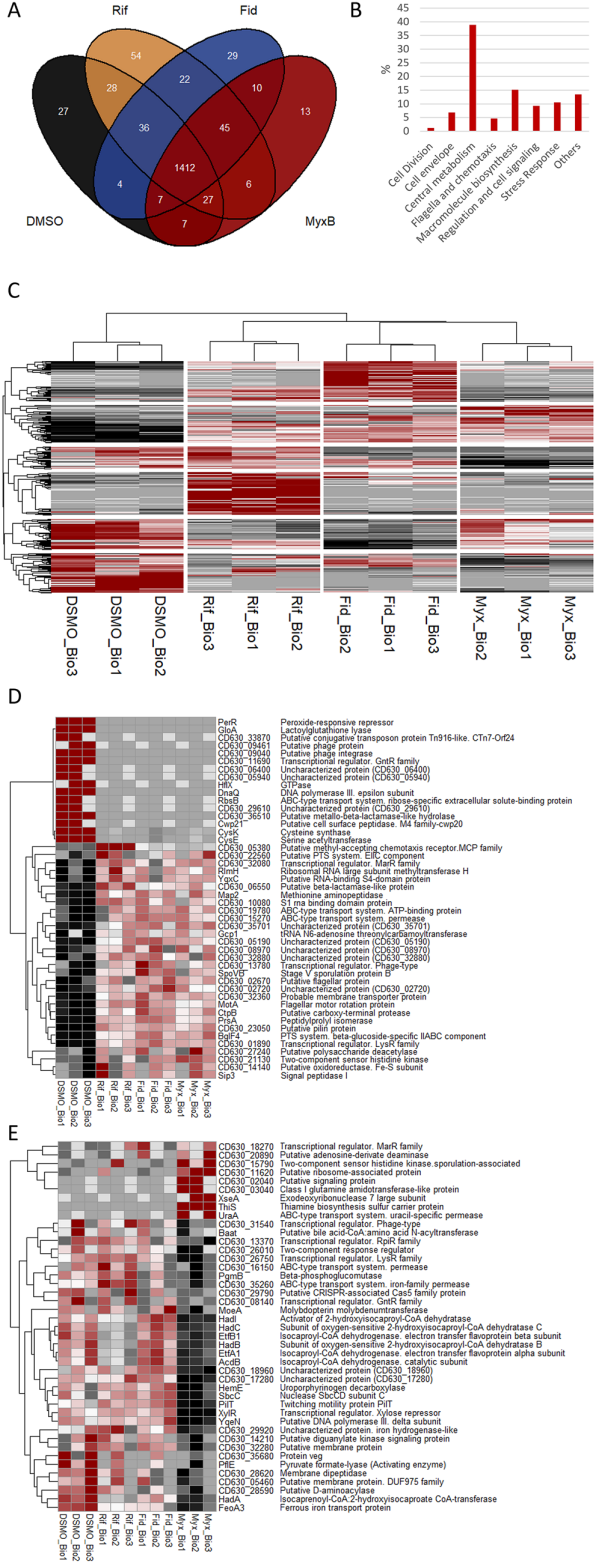
Differential protein abundance following Rifaximin, Fidaxomicin and Myxopyronin B stress in *C. difficile*. The protein inventory of *C. difficile* 630 after stress with sublethal concentrations of Rifaximin (1.75 ng/ml; Rif), Fidaxomicin (6 ng/ml; Fid) and Myxopyronin B (500 ng/ml, MyxB) was analyzed by LC–MS/MS. **A** Proteins identified with at least two unique peptides in at least two out of three biological replicates but not in the DMSO controls or *vice versa* are displayed in a Venn diagram drawn with the R package “ggvenn”. **B** Bar chart presenting the percentage of differentially abundant proteins associated with displayed metabolic functions. **C** A data subset including only proteins that were significantly altered in their abundance according to DEqMS analysis or were absent in at least one condition was analyzed by hierarchical clustering of z-transformed intensity-based quantitative data using the R package “pheatmap”. More or unique abundance of proteins following stress compared to the DMSO controls is indicated by red coloring, lower abundance or absence following stress by black coloring. **D** Heatmap displaying proteins homogenously more abundant or only identified after treatment with all three antibiotics. **E** Heatmap displaying proteins specifically more abundant or only identified after treatment with Myxopyronin B. *DMSO* DMSO-treated samples, *Rif* Rifaximin-treated samples, *Fid* Fidaxomicin-treated samples, *MyxB* Myxopyronin B-treated samples, *Bio1-3* biological replicates 1 to 3

To test for similarities and differences in the proteome stress signatures and proteins potentially associated with antimicrobial resistance to these antibiotics, a comprehensive LC–MS/MS analysis of the protein inventory of exponentially growing *C. difficile* 630 cells cultivated for 90 min in the presence of sublethal concentrations of Rifaximin, Fidaxomicin, and Myxopyronin B was performed. Thereby, between 1527 and 1631 *C. difficile* proteins could be identified in the four different conditions tested (Fig. 1A, see Additional file 1). 40, 57 and 10 were found to be significantly differentially expressed between Rifaximin-, Fidaxomicin or Myxopyronin B-treated cells and the DMSO controls, respectively. In addition, 82, 67 and 45 proteins were only identified in Rifaximin-, Fidaxomicin or Myxopyronin B-treated cells but not in the DMSO controls (Fig. 1A). Functional analysis revealed that most of these proteins are annotated as energy metabolism related proteins but also proteins required for macromolecule biosynthesis, stress response, regulation and cell signaling and other metabolic functions were among the differentially abundant proteins (Fig. 1B).

Hierachical cluster analysis of differentially abundant proteins revealed a similar stress response to all three antibiotics in *C. difficile* strain 630 (Fig. 1C). Several proteins from various functional categories such as, translation, flagella and membrane transport were found in higher amounts in response to all three antibiotics, while cysteine biosynthesis and some phage proteins showed lower abundances in the treated samples (Fig. 1D). However, some differences between the antibiotics signatures could be observed in various functional categories, such as energy metabolism, cell wall turnover, and vitamin synthesis (Fig. 1C, E, Additional file 1). For instance, most chemotaxis proteins were exclusively identified in Rifaximin-treated cells. In addition, proteins of the butyrate fermentation pathway were found in lower amounts in Rifaximin- and Fidaxomicin-treated cells while proteins from the branched chain amino acid fermentation pathway were lower abundant in *C. difficile* stressed with Myxopyronin B (Fig. 1C, E). However, as these effects are most likely off-target effects and not directly linked to antibiotic resistance mechanisms, they are not further discussed but can be completely reviewed in Additional file 1.

### Fidaxomicin and Myxopyronin B both hamper toxin synthesis

**Fig. 2:**
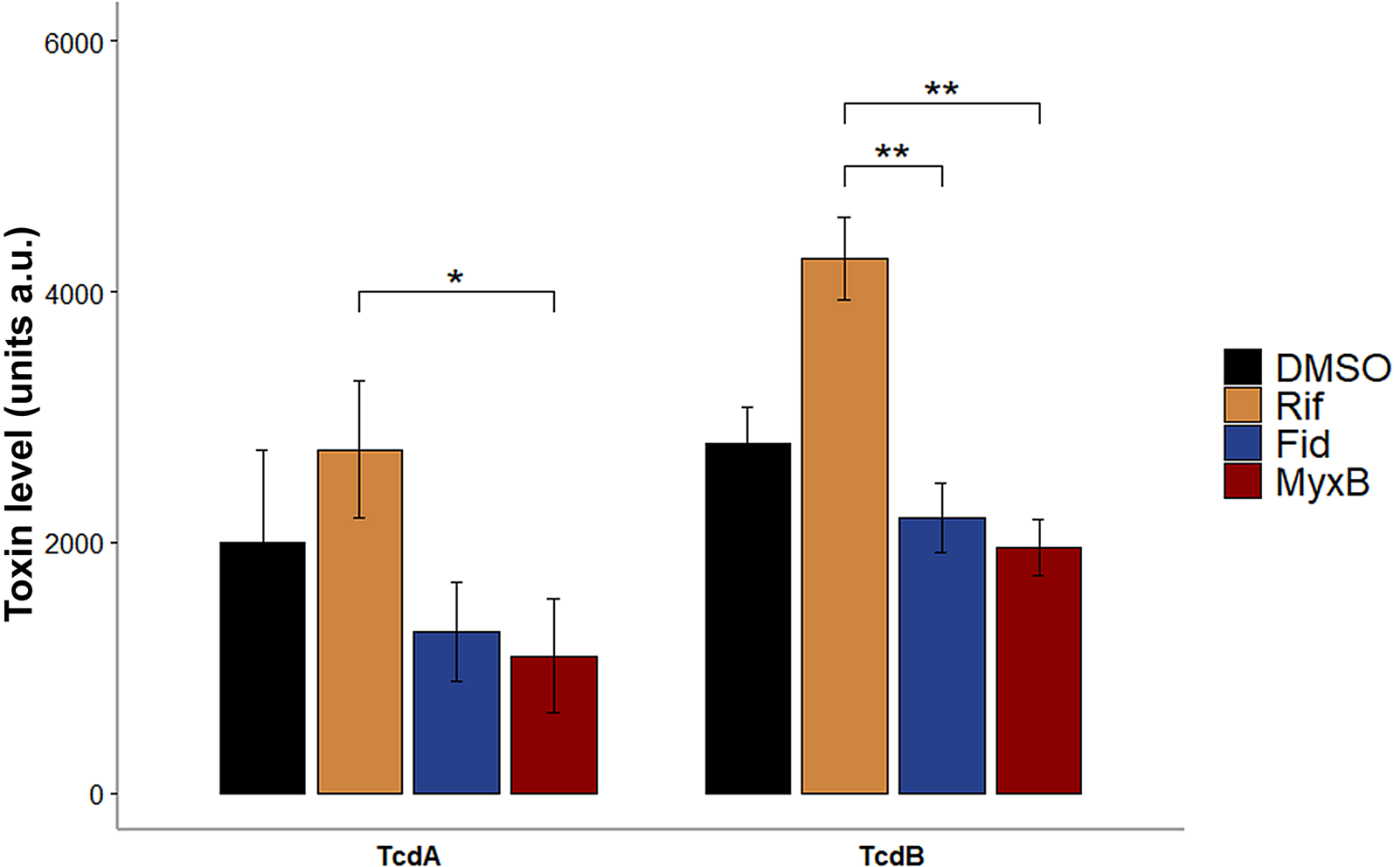
*C. difficile* toxin levels in the presence of sublethal concentrations of Rifaximin, Fidaxomicin and Myxopyronin B. Toxins A and B expression levels in *C. difficile* cells after stress with sublethal concentrations of Rifaximin (1.75 ng/ml; Rif), Fidaxomicin (6 ng/ml; Fid) and Myxopyronin B (500 ng/ml, MyxB) were quantified by western blot analysis. Values present the average signal intensities of toxin bands of three biological replicates in artificial units (a.u.) quantified by the “measure” tool of ImageJ. * indicates significant differences according to FDR adjusted t-testing using the R tool “rstatix”. *DMSO* DMSO-treated samples, *Rif* Rifaximin-treated samples, *Fid* Fidaxomicin-treated samples, *MyxB* Myxopyronin B-treated samples, *a.u.* artificial units

MS analysis revealed a lower abundance of toxin A in Fidaxomicin- and Myxopyronin B-treated cells compared to the control sample, while toxin A levels were higher in Rifaximin-treated cells compared to all other conditions (Additional file 2). Toxin B could not be detected via MS. To overcome this issue and to validate the observed effects on toxin synthesis, western blot analyses were performed in order to quantify toxins A and B. Indeed, the expressions of toxins A and B were both negatively affected by Fidaxomicin and Myxopyronin B but the abundance of toxins was higher in Rifaximin-treated cells, validating the MS results (Fig. 2, Additional file 3).

### Induction of proteins potentially involved in antibiotic tolerance

**Fig. 3:**
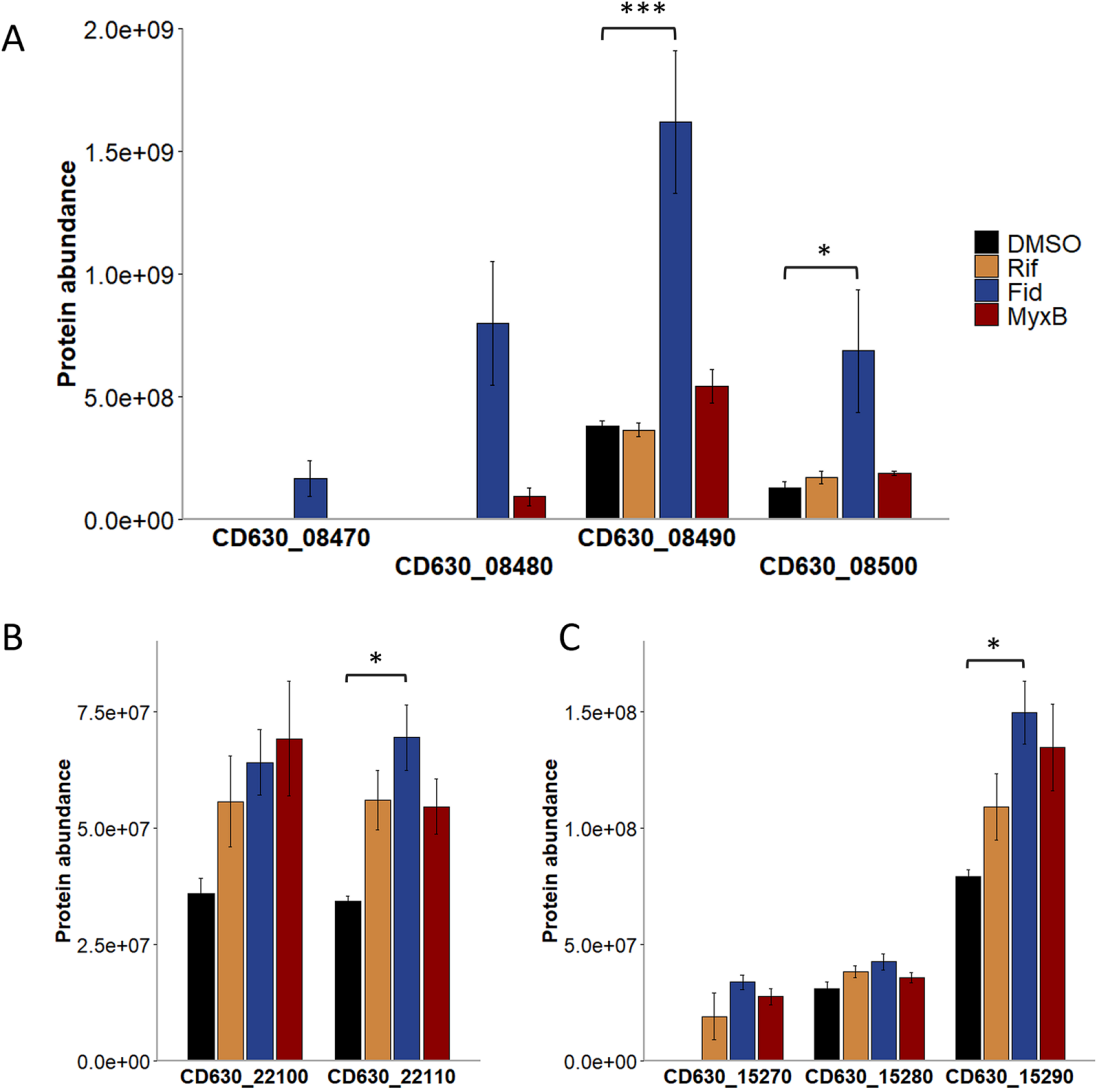
Differential abundance of selected proteins potentially associated with antibiotic tolerance in *C. difficile* after Rifaximin, Fidaxomicin and Myxopyronin B stress. Averaged relative protein intensities of three biological replicates of each individual protein are displayed and significant changes according to DEqMS analysis are indicated by a *. **A** Proteins of the *CD630_08470-CD630_08500* operon were induced in response to Fidaxomicin and to lesser extent to Myxopyronin B. **B** and **C** Two ABC transport system annotated as multidrug resistance efflux systems were induced in response to all three antibiotics. *DMSO* DMSO-treated samples, *Rif* Rifaximin-treated samples, *Fid* Fidaxomicin-treated samples, *MyxB* Myxopyronin B-treated samples

The MS data were then searched for proteins that exhibited different abundance between controls and treated samples and that consequently might be involved in antibiotic tolerance. Three operons could be identified, which seem to respond to Fidaxomicin and to lesser extent to the other two antibiotics. For instance, proteins which are encoded by the tetra-cistronic operon *CD630_08470-CD630_08500* were higher abundant in Fidaxomicin- and to lesser extent in Myxopyronin B-treated cells but not post Rifaximin administration compared to control cells (Fig. 3A). Whereas two proteins (CD630_08490 and CD630_08500) were significantly higher abundant in Fidaxomicin-treated cells but only slightly higher abundant in the presence of Myxopyronin B, the other two proteins were exclusively identified in Fidaxomicin- (CD630_08470 and CD630_08480) and Myxopyronin B-treated cells (CD630_08480). Moreover, two putative ABC antibiotic efflux associated proteins belonging to the *CD630_15290-CD630_15270* and *CD630_22120-CD630_22100* ABC transport systems were found in significantly higher amounts in Fidaxomicin-treated cells. A similar trend was observed in Rifaximin- and Myxopyronin B-treated cells although these effects were not significant (Fig. 3B and C).

## Discussion

Fidaxomicin is successfully used as an antibiotic for CDI therapy, and resistance rates of *C. difficile* to Fidaxomicin are still low [38]. Nevertheless, a first Fidaxomicin-resistant *C. difficile* isolate has been reported [21] and resistant strains could be obtained from laboratory experiments [39]. Therefore and for the sake of preparedness, alternative antibiotics for CDI therapy should be ready at hand. The data presented here suggest Myxopyronin B as a novel promising lead structure for the development of CDI antibiotics. Myxopyronins have been shown to be active against *C. difficile* [17] and based on structural analysis of antibiotic binding sites, cross-resistance between Rifamycins or Fidaxomicin and Myxopyronins is assumed to be unlikely [19, 23, 24]. Moreover, the frequency of antibiotic resistance to Myxopyronins was shown to be equal to that of Rifampin in *S. aureus*, but resistance to Myxopyronins proved to be associated with significant higher fitness costs reducing the risk of Myxopyronin resistance *in vivo* [40]. Although minimal inhibitory concentrations are comparatively high for Myxopyronin B against *C. difficile*, they are not influenced by mutations conferring Fidaxomicin resistance as shown in this study and inhibitory concentrations are lower than to those against selected anaerobic commensals from the gut. Furthermore, an early study reported that 100 mg/kg (applied subcutaneously) of Myxopyronins were tolerated in mice without acute toxicity [22]. Additionally, Myxopyronin B is able to suppress *C. difficile’s* toxin production under *in vitro* conditions in a similar manner as Fidaxomicin. While lower toxin synthesis in Fidaxomicin-treated cells has previously been reported [12], this is the first report on reduced toxin levels in Myxopyronin B-treated *C. difficile* cells. This observation, although validation is required, is of great importance since Fidaxomicin’s clinical efficacy is, among others, attributed to its ability to control *C. difficile’s* pathogenicity [13]. While higher toxin levels in early phase Rifaximin-treated cells might be part of the general stress response of *C. difficile* and have been observed for other antibiotics before [41–43], the reduced abundance of toxins in response to Fidaxomicin is not completely understood. Overall, the complex regulatory circuits underlying toxin synthesis in *C. difficile* involve several regulatory proteins, are connected to numerous environmental stimuli and are in part also strain-dependent [44]. However, toxin synthesis has been linked to transcription arrest and damage via the SOS response regulator LexA and the transcription repair coupling factor Mfd [45, 46]. Therefore, high toxin levels in Rifaximin-treated cells are likely the result of transcription arrest. In contrast, both Fidaxomicin and Myxopyronin B potentially avoid derepression of toxin synthesis by interruption of transcription at an earlier timepoint and, in turn, circumvent activation of repair and stress response systems. Despite the lack of knowledge regarding the underlying regulatory mechanisms, the low toxin levels in Myxopyronin B-treated cells suggest that patients receiving Myxopyronin B might potentially benefit from Myxopyronin B treatment in a similar way as reported for Fidaxomicin. Further experiments will be required to prove these initial results, which might include determination of *tcdA* and *tcdB* mRNA levels in response to Myxopyronin B or the analysis of toxin production in gut model systems resembling infection conditions. Similarly, the Myxopyronin tolerance of other intestinal bacteria and of complex intestinal communities needs to be analyzed more comprehensively by a meta-omics approach to validate initial data presented here. Finally, pharmaceutical engineering, although requiring time and effort, could further optimize activity, stability and solubilty of the compound.

Transcriptome and proteome signatures of antibiotic-stressed bacterial cells are a valuable tool to obtain insights in the cellular effects that are induced by a respective antibiotic and therefore provide a good starting point to characterize new antibiotics and estimate their potential as antiinfective drug [47–49]. For instance, a recent publication on various antibiotic signatures in *B. subtilis* revealed a number of marker proteins, which are shared between antibiotics with a similar mode-of-action and allowed to draw hypotheses on the mode-of-action of hitherto uncharacterized antibiotics [49]. However, effects observed on transcriptome and proteome level in response to a specific antibiotic may vary depending on the bacterial strain and the experimental set up, comprising factors such as antibiotic concentration, harvest timepoints, sample preparation and analysis. In turn, comparability between studies should be stronger for a core set of cellular pathways which most likely comprise the direct effects of an antibiotic while secondary and off-target effects might vary depending on the experimental set up [50–55]. Such secondary effects always accompany antibiotic stress [56, 57] and might be the consequence of a general growth retardation, changing membrane permeability or the requirement for metabolites needed to deal with the antibiotic stress [58–60]. Consequently, it is of great value to compare the stress signatures of antibiotics in a defined experimental set up to allow sufficient comparability, as it was done in this study. Hierachical cluster analysis of proteome stress signatures of the three RNA polymerase inhibitors revealed a subset of proteins, which show a similar trend. Since these cluster comprise transcription and translation-associated proteins, clustering most likely reflects the shared mode-of-action, i. e. interfering with the transcription process [61, 62]. Despite different strains used (630 *vs.* 630Δ*erm*), different harvest timepoints (90 min *vs.* 10 and 30 min) and different methods (label-free LC–MS/MS *vs.* pulsed-labeling followed by 2D-based MALDI-TOF MS), a recently published Fidaxomicin-stress signature in *C. difficile* 630Δ*erm* revealed similar differentially expressed proteins involved in transcription, protein biosynthesis, nucleotide metabolism and motility [61].

In contrast, several other marker proteins obtained from cells treated with Fidaxomicin for 10 and 30 min as part of the pulsed-chase experiment conducted by Maaß et al. [61] were not differentially expressed in our study and vice versa, which can most likely be attributed to the different experimental setups of the two studies. Maaß et al. also investigated *C. difficile*’s stress response to antibiotics, e. g. Metronidazole and Vancomycin, targeting other cellular structures than RNA polymerase [61]. The stress response patterns clearly differ from the one of Fidaxomicin affirming the feasibilty of comparative proteomics to uncover cellular targets of antimicrobial substances.

Differences observed for the three RNA polymerase signatures presented in this study, such as those related to the energy metabolism, most likely present secondary effects to adapt to changing conditions. For example, proteins for leucine fermentation were detected in lower amounts in Rifaximin- and Myxopyronin B-treated cells but not in Fidaxomicin-treated cells, while proteins from the butyrate fermentation operon were only lower abundant in response to Fidaxomicin. Such off-target effects most likely also contribute to the antibiotic’s antimicrobial activity and have been reported before, e.g. for Rifampin [63]. However, their precise role in antibiotic-mediated killing by a respective antibiotic requires further validation.

A few other observations in the proteome stress signatures caught our attention. Considering their potential role in antibiotic tolerance in *C. difficile,* they should be considered as a starting point for future analyses. First of all, proteins of the *CD630_08470-CD630_08500* operon were found to be elevated after Fidaxomicin stress and to a lesser extent after Myxopyronin B stress. The role of this operon in *C. difficile* is unknown. However, *CD630_08490*, the third gene of the operon, is annotated as AbgA, an aminobenzoylglutamate utilization protein, which was shown to be repressed by CodY [64]. Aminobenzoylglutamate utilization proteins are required to hydrolyze *p*-aminobenzoyl-glutamate to folate which is, in turn, required for DNA and RNA synthesis [65]. The last gene of the operon, *CD630_08500*, encodes for a NifU-like protein. Nif system proteins are involved in the formation of FeS clusters [66]. The remaining two genes of the operon, *CD630_08470* and *CD630_08480* are not annotated and neither a BLASTp analysis nor a literature search provided information on their function. AbgA is linked in many species to the aminobenzoylglutamate transport protein AbgT [67] which is encoded by *CD630_28350* in *C. difficile* 630 but could not be identified in the proteome data. Interestingly, the function of the AbgT transporter family has recently been revised and it is now linked to sulfonamide resistance by functioning as an efflux system for the export of sulfonamide antibiotics [67]. Induction of the *CD630_08470-CD630_08500* operon in response to Fidaxomicin and to a lesser extent in response to Myxoypronin B might provide folate for DNA/RNA synthesis but may also be linked to antibiotic resistance.

Antibiotic efflux is known to be a major contributor to antibiotic resistance in many pathogens [68]. However, only a few antibiotic efflux systems have been characterized in detail in *C. difficile* [69–72]. ABC type antibiotic efflux systems might either be specific for a class of compounds or can transport a large group of compounds. Thus, they can significantly contribute to antibiotic resistance of a pathogen or be of minor relevance [68, 70, 72]. Elevated levels of proteins from the two ABC transport systems *CD630_22120-CD630_22100* and *CD630_15290-CD630_15270*, annotated as multidrug-resistance transporters, after stress with all three RNA polymerase inhibitors suggest their potential role in the response to transcription inhibition or to antibiotic stress in general. However, further studies will be necessary to unravel the precise function of the respective systems.

## Conclusion

In summary, the presented study successfully proved the antimicrobial activity of Myxopyronin B against a Fidaxomicin-resistant isolate of *C. difficile* and provided the first stress signature for Myxopyronins. Moreover, a negative effect of Myxopyronin B on *C. difficile’s* toxin production and a low sensitivity of other anaerobes is supported by the presented data. Although attempts are ongoing to modify Fidaxomicin to provide novel compounds that overcome resistance mechanisms [73] and several other candidate antibiotics are being investigated [74], the results of this study, in concert with the potential fitness costs associated with Myxopyronin B resistance mutations [40], highlight Myxopyronin B as a promising new candidate antibiotic for CDI. In addition, our data suggest a potential role of a hitherto uncharacterized operon *CD630_08470-CD630_08500* and of two efflux systems (*CD630_15290-CD630_15270* and *CD630_22120-CD630_22100*) in the tolerance to RNA polymerase inhibitors or antibiotics in general.

## Supplementary Information

**Additional file 1**. Log2 fold changes and adjusted p values from DEqMS analyses for Rifaximin (Rif), Fidaxomicin (Fid) and Myxopyronin B (MyxB) treated *C. difficile* cells compared to control cells only exposed to DMSO (ctrl). Columns A to C and J to K give additional information on the proteins obtained from UniProt or microbes online. LFQ intensities and averaged LFQ intensities of all three biological replicates are given in columns L to AA. Significant p values are highlighted in red. Proteins only identified in stressed cells are listed as "ON" while proteins only identified in controls are listed as "OFF".

**Additional file 2**. Toxin A levels in *C. difficile* cells after stress with sublethal concentrations of Rifaximin (1.75 ng/ml; Rif), Fidaxomicin (6 ng/ml; Fid) and Myxopyronin B (500 ng/ml, MyxB) on LC-MS/MS level.

**Additional file 3**. Western blot images for quantification of toxin levels in *C. difficile* cells after stress with sublethal concentrations of Rifaximin (1.75 ng/ml; Rif), Fidaxomicin (6 ng/ml; Fid) and Myxopyronin B (500 ng/ml, MyxB) using antibodies against toxin A (left) and toxin B (right). Per condition, three biological replicates were analyzed. The additional band in the toxin A blot at appr. 150 kDa represents a protein which is unspecifically bound by the primary toxin A antibody.

## Data Availability

The mass spectrometry proteomics data have been deposited to the ProteomeXchange Consortium via the PRIDE partner repository with the dataset identifier PXD027366.

## Abbreviations

a.u.: Artificial units
CDI: *Clostridioides* *difficile* Infections
Fid: Fidaxomicin
MS: Mass spectrometry
MyxB: Myxopyronin B
Rif: Rifaximin
RT: Ribotype
