# Supplementary figures and images for: Myxopyronin B inhibits growth of a Fidaxomicin-resistant Clostridioides difficile isolate and interferes with toxin synthesis
Source: Gut Pathog. 2022 Jan 6;14:4. doi: 10.1186/s13099-021-00475-9 (PMC8739712; doi:10.1186/s13099-021-00475-9)

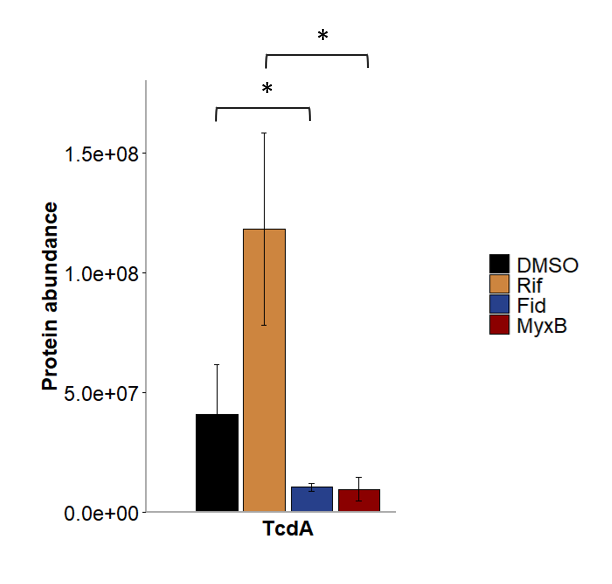

Supplement: Supplementary file 2 — Additional file 2. Toxin A levels in C. difficile cells after stress with sublethal concentrations of Rifaximin (1.75 ng/ml; Rif), Fidaxomicin (6 ng/ml; Fid) and Myxopyronin B (500 ng/ml, MyxB) on LC-MS/MS level. [file 13099_2021_475_MOESM2_ESM.png]

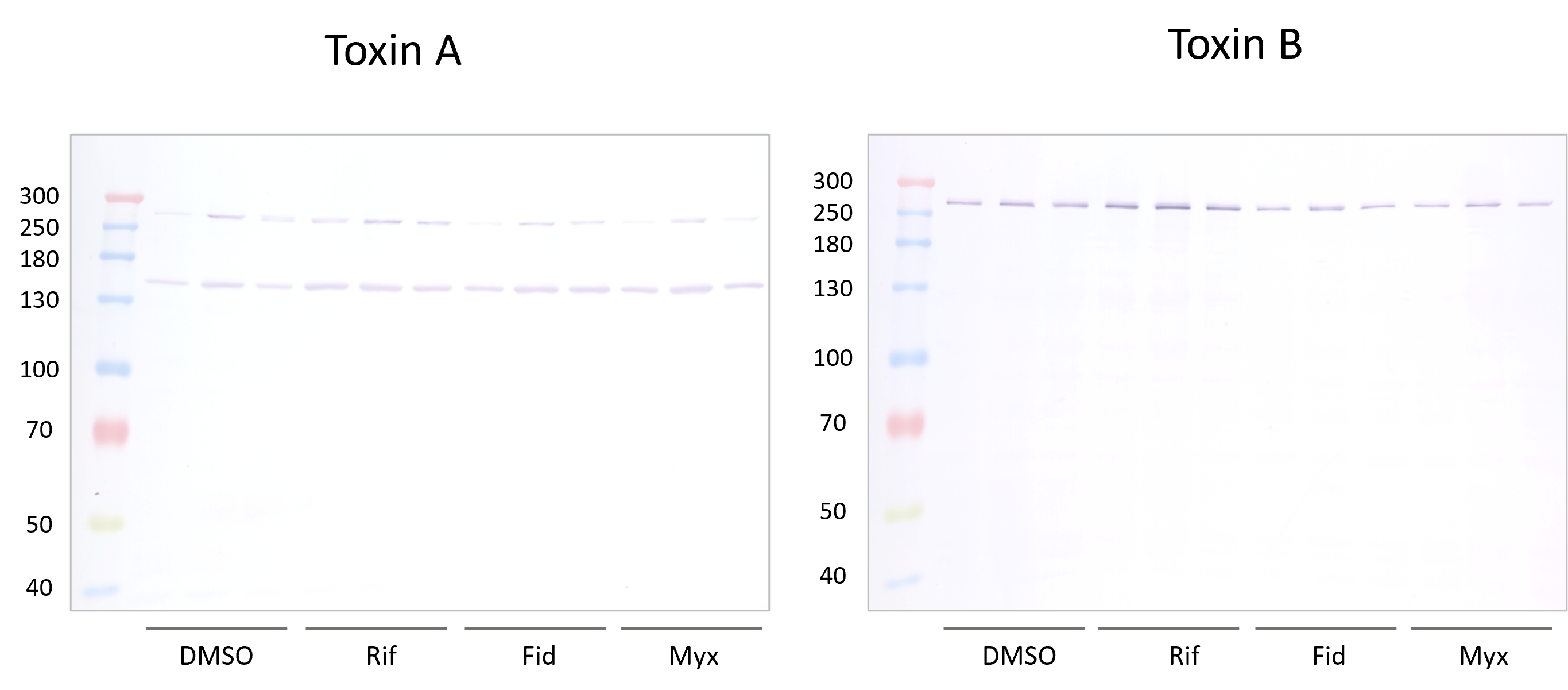

Supplement: Supplementary file 3 — Additional file 3. Western blot images for quantification of toxin levels in C. difficile cells after stress with sublethal concentrations of Rifaximin (1.75 ng/ml; Rif), Fidaxomicin (6 ng/ml; Fid) and Myxopyronin B (500 ng/ml, MyxB) using antibodies against toxin A (left) and toxin B (right). Per condition, three biological replicates were analyzed. The additional band in the toxin A blot at appr. 150 kDa represents a protein which is unspecifically bound by the primary toxin A antibody. [file 13099_2021_475_MOESM3_ESM.png]
